# Clinical Practice Guideline for Management of Primary Aldosteronism: What is New in the 2016 Update?

**DOI:** 10.16966/2380-548X.129

**Published:** 2016-07-11

**Authors:** Damian G Romero, Licy L Yanes Cardozo

**Affiliations:** 1 Department of Biochemistry, University of Mississippi Medical Center Jackson, USA; 2 Women’s Health Research Center, University of Mississippi Medical Center and Jackson, USA; 3 Cardio-Renal Research Center, University of Mississippi Medical Center Jackson, USA; 4 Department of Physiology and Biophysics, University of Mississippi Medical Center Jackson, USA; 5 Department of Medicine, University of Mississippi Medical Center Jackson, USA

**Keywords:** Primary Aldosteronism, Adrenocorticotrophic hormone, Aldosterone-producing adenoma

## Abstract

Primary Aldosteronism is the single most common cause of secondary hypertension and is associated with increased target organ injury. The Endocrine Society has recently released the updated Clinical Practice Guideline for Primary Aldosteronism entitled “The Management of Primary Aldosteronism: Case Detection, Diagnosis, and Treatment: An Endocrine Society Clinical Practice Guideline”. We review the updated Clinical Practice Guideline, highlighting the new recommendations and the implications that they may have in clinical practice. The recognition by the Endocrine Society’s Task Force that Primary Aldosteronism is a public health issue and that the population at risk for screening should be significantly expanded will surely have an impact in the clinical practice which hopefully will translate in better detection, diagnosis and treatment of patients with Primary Aldosteronism.

## Introduction

The Endocrine Society has recently released the updated Clinical Practice Guideline for Primary Aldosteronism (PA) entitled “The Management of Primary Aldosteronism: Case Detection, Diagnosis, and Treatment: An Endocrine Society Clinical Practice Guideline” [1]. The updated guideline includes both specific new recommendations for case detection, diagnosis, and treatment of PA as well as more profound philosophical changes due to our current better understanding of this disease.

The steroid hormone aldosterone is the main mineralocorticoid and is synthetized by the zona glomerulosa of the adrenal gland cortex [2,3]. The main physiological regulators of aldosterone synthesis and secretion are angiotensin II (the end product of the renin-angiotensin system), plasma potassium and the adrenocorticotrophic hormone (ACTH), beside a plethora of regulators that fine-tune aldosterone secretion under different physiological conditions [4]. Aldosterone binds to the renal mineralocorticoid receptor (MR) to exert its classical biological action stimulating sodium reabsorption and potassium excretion modulating gene expression in target cells [5,6]. Besides aldosterone classical epithelial actions that translate in the regulation of extracellular volume and blood pressure, the mineralocorticoid also exerts multiple biological actions in non-epithelial tissues [5]. Autonomous excess aldosterone synthesis and secretion is the hallmark of PA which is characterized by hypertension, and target organ injury and dysfunction, with the latest ones being even worse than the ones observed in hypertensive patients from other etiologies than PA [7-10].

The Clinical Practice Guideline for PA was first published by the Endocrine Society in 2008 [11]. Eight years later and with a substantial increase of our understanding on susceptible populations, prevalence, genetics, target-organ damage and therapies for PA, the 2008 Guideline [11] obviously needed an update. For that purpose, the Endocrine Society commissioned a Task Force composed of eight experts on PA to address this demand and generated the updated Guideline [1]. The updated Guideline not only include experts in the Task Force from four continents but it is also sponsored by scientific societies from all around the world including American Heart Association, American Association of Endocrine Surgeons, European Society of Endocrinology, European Society of Hypertension, International Association of Endocrine Surgeons, International Society of Endocrinology, International Society of Hypertension, Japan Endocrine Society, and The Japanese Society of Hypertension.

Furthermore, the Task Force did not receive any corporate funding or remuneration to keep it as unbiased as possible.

We review the Clinical Practice Guideline for PA emphasizing what is new in the updated one.

## What is Primary Aldosteronism?

Primary Aldosteronism (PA) is defined as a group of disorders in which aldosterone production is inappropriately high for sodium status, relatively autonomous of the major regulators of secretion (angiotensin II, plasma potassium concentration), and nonsuppressible by sodium loading. Excess aldosterone, inappropriately high for the salt intake status, causes hypertension, cardiovascular and renal damage, sodium retention, suppression of plasma renin, and increased potassium excretion that (if prolonged and severe) may lead to hypokalemia. PA is commonly caused by an adrenal adenoma, unilateral or bilateral adrenal hyperplasia, or in rare cases adrenal carcinoma or inherited conditions of familial hyperaldosteronism.

It is critical to keep PA definition in mind since it will guide us in the analysis of PA case detection, diagnosis, and treatment.

## How common is Primary Aldosteronism?

Multiple epidemiological studies in the last two decades have shown that PA has a prevalence of >5% (possible even >10%) of hypertensive patients, both in general and specialty settings [12]. First described in the mid 1950’s [13] and although reported by Jerome Conn as representing a significant fraction of hypertensive patients [14], PA went under the radar in the following decades, not because it was not present, but due to screening deficiencies relying in hypokalemia as a PA hallmark. Latter studies showed that hypokalemia is present in only~10-40% of patients with PA [15,16]. With better screening methods and not relying in the assumption that hypokalemia is a sine qua non hallmark of PA, a series of epidemiological studies have clearly shown that PA is much more common than previously thought [12]. The guidelines are a call for physicians to realize that PA patients are a significant proportion of their daily hypertension-related consults. If we assume, in general terms, that the worldwide incidence of hypertension is~22% [17] and between 5-10% of them suffer from PA [12], we can conclude that between 1.1% and 2.2% of the general population suffers from PA. These impressive prevalence numbers are the driving forces that probably lead the Task Force to declare PA as a major health care issue that impacts not only the individual but the society as a whole.

## How Frequent is Hypokalemia in Primary Aldosteronism?

Nowadays, nobody doubts that the main physiological effect of aldosterone in the kidney is to increase sodium reabsorption and potassium excretion. It is intuitive to think that an excess aldosterone, such as in PA, will cause an increased potassium excretion leading to hypokalemia. However, that is not the case in the majority of PA cases. Several epidemiological studies have shown that hypokalemia is present in only~10-40% of patients with PA [15,16]. The cause for the lack of hypokalemia in PA, particularly in the less florid cases, is not clear but what it is absolutely clear nowadays is that the use of hypokalemia as a PA screening test should be discontinued.

## Why we should Care about Primary Aldosteronism Diagnosis?

PA is the single most common cause of secondary hypertension. Moreover, PA patients have higher cardiovascular morbidity and mortality than patients with essential hypertension with similarly elevated blood pressure [18-21]. Consequently, PA is much more than another cause of hypertension and targeted specific therapies, either surgical or medical, is preferred over non-specific blood pressure reducing therapies.

## Who should be screened for Primary Aldosteronism?

Screening for PA is recommended for subjects who meet one of the following criteria:

- Subjects with sustained blood pressure (BP) above 150/100 mm Hg on each of three measurements obtained on different days;
- Subjects with hypertension (BP>140/90 mm Hg) resistant to three conventional antihypertensive drugs (including a diuretic);
- Subjects with controlled BP (<140/90 mm Hg) on four or more antihypertensive drugs;
- Subjects with hypertension and spontaneous or diuretic-induced hypokalemia;
- Subjects with hypertension and adrenal incidentaloma;
- Subjects with hypertension and sleep apnea;
- Subjects with hypertension and a family history of early onset hypertension or cerebrovascular accident at a young age (<40 years); and
- All hypertensive first-degree relatives of patients with PA.

The first category (“Subjects with sustained blood pressure above 150/100 mm Hg”) is an addition to the updated Guideline in line with the recognition that PA is common (5-10%) between hypertensive patients and should no longer be considered a rare disease among them.

The Task Force recognizes that any intervention, including diagnosis procedures are not free of adverse events. However, overall, the benefits of PA diagnosis (and treatment) overcome the risk associated with diagnosis procedures in subjects negative for PA. However, this assumption is yet to be proven. It will take decades to know, if broadening the population at risk for screening as much as suggested turns out to be as beneficial for public health as expected.

## What test should be used to screen for Primary Aldosteronism?

The gold standard for PA case detection is the plasma aldosterone/ renin ratio (ARR) as it is currently the most reliable test for PA screening. However, it is critical to interpret the ARR in the context of the plasma aldosterone concentration (PAC) value. An elevated ARR value with a low PAC is probably indicative of an ARR value artificially elevated due to a very low plasma renin activity (PRA) or plasma renin concentration that is not suggestive of PA as the cause of the abnormality. Clinical laboratories are encouraged to always report, and physicians always interpret, ARR as well as PAC and, PRA or plasma renin concentration.

The updated Guideline [1] states rigorous procedures for accurate ARR sample collection and analysis as well as a series of interfering factors in the ARR results that are very important to keep in mind to avoid both false positives or negatives.

## Should ARR-identified cases of Primary Aldosteronism be confirmed?

Short answer is “yes”; although, no “gold standard” confirmatory test for PA has been suggested. The most common PA confirmatory tests are:

- Oral sodium loading test;
- saline infusion test;
- fludrocortisone suppression test; and
- captopril challenge test.

PA confirmatory test selection should be done to maximize efficacy, cost, patient compliance, laboratory routine, and should ideally be interpreted by individuals with expertise in those tests such as endocrinologists.

An exception to the confirmatory test suggestion is in the case of subjects with spontaneous hypokalemia, plasma renin below detection levels plus PAC>20 ng/dL (550 pmol/L). Under these conditions, PA is the most likely diagnosis and no further confirmatory testing is necessary. Essentially, if a PA patient passes the “duck test” for PA (“If it looks like a duck, swims like a duck, and quacks like a duck, then it probably is a duck”) it is probably PA.

## Is Primary Aldosteronism Subtype classification Necessary?

PA subtype classification is critical as it will guide future patient management. Unilateral PA should optimally be treated by laparoscopic adrenalectomy; while, bilateral adrenal hyperplasia or patients unsuitable for surgery should be treated primarily with a mineralocorticoid receptor (MR) antagonist.

## How does Primary Aldosteronism subtype classification should be done?

It is recommend that all patients with PA undergo adrenal computed tomography (CT) as the initial study in subtype testing to exclude large masses that may represent adrenocortical carcinoma and to assist the interventional radiologist and surgeon where anatomically appropriate. However, adrenal carcinoma cases presenting with clinical features of PA are rare. As a consequence of the updated Guideline, we will probably observe a significant increment in the number of CTs to be performed. A down the road cost-benefit analysis of this suggestion to perform a CT to each detected case of PA would be critical to refine this Guideline in the future.

If surgical treatment is feasible and desired by the patient, an experienced radiologist should use adrenal venous sampling (AVS) to make the distinction between unilateral and bilateral adrenal disease. AVS is the “gold standard” test to distinguish unilateral (aldosterone-producing adenoma [APA] or unilateral adrenal hyperplasia [UAH]) from bilateral (idiopathic adrenal hyperplasia [IAH]) disease in patients with PA. However, it is important to stress that AVS is a difficult and technically demanding procedure that is highly dependent on the operator. AVS may be performed under unstimulated or Cosyntropin-stimulated (either, bolus or continuous infusion) conditions.

## What is the Recommended Treatment for Primary Aldosteronism?

Confirmed PA cases should be managed according with PA subtype and surgery feasibility and willingness by the patient.

In cases of unilateral PA (i.e., APA or UAH), laparoscopic adrenalectomy is the treatment of choice. Adrenalectomy is highly effective in normalizing plasma aldosterone and potassium levels as well as reducing blood pressure and cardiac hypertrophy and fibrosis. If a patient is unable or unwilling to undergo surgery, it is recommend medical treatment including a MR antagonist (MRA). Furthermore, if an ARR-positive patient is unwilling or unable to undergo further investigations, it is strongly recommend that medical treatment including an MRA be pursued.

In cases of bilateral PA (i.e., IAH), medical treatment with an MRA is recommended. Moreover, spironolactone is recommended as the primary agent, with eplerenone as an alternative choice. Due to differences in half life, spironolactone is dosed once daily while eplerenone is dosed twice daily.

The MRAs currently available in the market are spironolactone and eplerenone [22,23]. First-generation MRA spironolactone is a potent MRA but lacks selectivity, binding also to the androgen and progesterone receptors, which translates in the classical adverse effect of gynecomastia. On the other hand, second-generation MRA eplerenone is a much more selective MRA but less potent and more expensive than spironolactone. The mechanism of action of both MRAs is competitive inhibition of aldosterone binding to the MR blocking MR-mediated gene expression regulation. Hyperkalemia is the most serious and life threatening main adverse effect of both MRAs; consequently, close monitoring of plasma potassium levels is strongly advised [22,24]. Both, aldosterone synthase inhibitors and third-generation non-steroidal MRAs, are currently in clinical trials and may be better therapeutic options in the future [23,25,26].

A new recommendation of the Guideline is that younger patients (age<35 years) with hallmark characteristics of PA such as spontaneous hypokalemia, marked aldosterone excess, and unilateral adrenal lesions with radiological features consistent with a cortical adenoma on adrenal CT scan may not need AVS before proceeding to unilateral adrenalectomy.

## Are there Any Recommendations for Familiar Forms of Primary Aldosteronism?

Familial hyperaldosteronism type 1 (FH-I) also known as glucocorticoid remediable aldosteronism (GRA) is an inheritable form of PA accounting for~1% cases of PA. FH-I is caused by fusion of the promoter region of the gene for 11β-hydroxylase (CYP11B1) and the coding sequences of aldosterone synthase (CYP11B2), resulting in a CYP11B1/CYP11B2 chimeric gene. FH-I is a form of PA in which the hypersecretion of aldosterone is dependent upon endogenous ACTH secretion which leads to an increase in aldosterone synthesis.

In patients with an onset of confirmed PA earlier than 20 years of age and in those who have a family history of PA or strokes at a young age (<40 years), it is suggested genetic testing for FH-I (GRA). FH-I diagnosis is critical since this type of PA requires a completely different treatment that deals with the genetic rearrangements that cause it. In patients with GRA, it is recommended the administration of the lowest dose of glucocorticoid to lower ACTH and thus normalize blood pressure and potassium levels as the first-line treatment. Furthermore, if blood pressure is not normalized with glucocorticoid alone, a combinatorial therapy with an MR antagonist is suggested.

Familial hyperaldosteronism type 2 (FH-II) is a genetically heterogeneous autosomal dominant disorder for which not specific diagnosis or treatment recommendations are given.

Familial hyperaldosteronism type 3 (FH-III) is caused by mutations the KCNJ5 gene encoding the potassium channel Kir 3.4 (potassium inwardly rectifying channel, subfamily 1, member 5). Although the genetic cause of FH-III is known, there are not targeted therapies currently available for KCNJ5 mutations and consequently standard surgical or medical therapies used for other cases of PA are recommended. Despite the lack of targeted therapies for FH-III, in very young patients with PA it is recommended to test for germline mutations in KCNJ5 for genetic counseling.

## Are there Genetic causes of Non-Hereditary Cases of APA?

For decades, the molecular basis of sporadic (non-hereditary) PA cases was unknown. However, in the last five years, mutations in a series of genes have been reported and epidemiological studies have shown that these mutations are responsible for~50% of the cases of sporadic PA. KCNJ5 gene, the same gene associated with FH-III, has been extensively scrutinized and more than 15 mutations have been reported associated with PA. Other genes, such as ATP1A (α subunit of the Na^+^/K^+^-ATPase), ATP2B2 (plasma membrane Ca^2+^-ATPase isoform 3), CACNA1D (α1D subunit of a voltage-gated L-type calcium channel) and CTNNB1 (β-catenin) [27].

Furthermore, other genes such as CACNA1H, ARMC5, LGR5, DACH1, NCAM1, GRM3, PCP4 and VPREB3 have been implicated as putative causes of PA. However, although exciting, the detection of such mutations do not have current clinical implications but may have in no such a distant future.

## Summary

The updated Clinical Practice Guideline for PA [1] have new recommendations for PA case detection, diagnosis and treatment but also, and equally important, emphasize concepts that we already know but usually forgot. PA is a fairly common disease that is not benign and the prognosis of undiagnosed or untreated PA patients is far worse than for other hypertensive patients. PA diagnosis and treatment should not be delayed. If PA confirmation or subtype classification is difficult due to lack of resources, medical treatment with affordable mineralocorticoid antagonists such as spironolactone should not be delayed.

Probably, the recommendation with broader and more profound implications will be the one to expand screening for PA to all patients with sustained blood pressure above 150/100 mm Hg; which is in agreement with the acknowledgment that PA is a public health issue.

Physicians and health care providers need to have a cultural change because it is unacceptable that nowadays, where and when resources are available, PA patients go untreated because we are not willing to accept that PA is a public health burden.

## Abbreviations

AAR: Aldosterone/Renin Ratio

ACTH: Adrenocorticotrophic Hormone

APA: Aldosterone-Producing Adenoma

ATP1A: α subunit of the Na^+^/K^+^-ATPase

ATP2B2: plasma membrane Ca^2+^-ATPase isoform 3

AVS: Adrenal Venous Sampling

BP: Blood Pressure

CACNA1D: α1D subunit of a voltage-gated L-type calcium channel

CT: Computed Tomography

CTNNB1: β-catenin

CYP11B1: 11β-hydroxylase

CYP11B2: aldosterone synthase

FH-I: Familial Hyperaldosteronism type 1

FH-II: Familial Hyperaldosteronism type 2

FH-III: Familial Hyperaldosteronism type 3

GRA: Glucocorticoid Remediable Aldosteronism

IAH: Idiopathic Adrenal Hiperplasia

Kir 3.4: potassium inwardly rectifying channel, subfamily 1, member 5

MR: Mineralocorticoid Receptor

MRA: Mineralocorticoid Receptor Antagonist

PA: Primary Aldosteronism

PAC: Plasma Aldosterone Concentration

UAH: Unilateral Adrenal Hyperplasia
